# Method for Dual Viral Vector Mediated CRISPR-Cas9 Gene Disruption in Primary Human Endothelial Cells

**DOI:** 10.1038/srep42127

**Published:** 2017-02-15

**Authors:** Haixia Gong, Menglin Liu, Jeff Klomp, Bradley J. Merrill, Jalees Rehman, Asrar B. Malik

**Affiliations:** 1 Department of Pharmacology, University of Illinois College of Medicine, Chicago, IL 60612, USA; 2 The Center for Lung and Vascular Biology, University of Illinois College of Medicine, Chicago, IL 60612, USA; 3 Department of Biochemistry and Molecular Genetics, University of Illinois College of Medicine, Chicago, IL 60612, USA; 4 Genome Editing Core, University of Illinois College of Medicine, Chicago, IL 60612, USA; 5 Division of Cardiology, Department of Medicine, University of Illinois College of Medicine, Chicago, IL 60612, USA

## Abstract

Human endothelial cells (ECs) are widely used to study mechanisms of angiogenesis, inflammation, and endothelial permeability. Targeted gene disruption induced by Clustered Regularly Interspaced Short Palindromic Repeats (CRISPR)-CRISPR-Associated Protein 9 (Cas9) nuclease gene editing is potentially an important tool for definitively establishing the functional roles of individual genes in ECs. We showed that co-delivery of adenovirus encoding EGFP-tagged Cas9 and lentivirus encoding a single guide RNA (sgRNA) in primary human lung microvascular ECs (HLMVECs) disrupted the expression of the Tie2 gene and protein. Tie2 disruption increased basal endothelial permeability and prevented permeability recovery following injury induced by the inflammatory stimulus thrombin. Thus, gene deletion via viral co-delivery of CRISPR-Cas9 in primary human ECs provides a novel platform to investigate signaling mechanisms of normal and perturbed EC function without the need for clonal expansion.

Endothelial cells (ECs), lining the intima of all blood and lymphatic vessels, regulate vessel formation through angiogenesis and lymphangiogenesis as well as the functions of normal and diseased vessels^1^. Primary cultures of human ECs are widely used to study cancer metastasis, adhesion and transmigration of leukocytes, wound healing, interaction of clotting factors with endothelium, and vascular barrier integrity^2^,^3^. The primary approach thus far has been to knockdown expression of specific genes in ECs using siRNA or shRNA constructs^4^,^5^. However, these approaches are limited by transient or incomplete gene depletion and off-target effects^6^. In the present report, we describe the first use of CRISPR-Cas9 to delete genes of interest in primary cultures of human ECs without clonal expansion using dual viral vectors.

Engineered type II CRISPR-CRISPR-associated protein 9 nuclease (Cas9) is widely accepted as a tool to rapidly and efficiently manipulate genomes of mammalian cells^7^,^8^,^9^. Cas9 can be targeted to defined DNA sequences by single guide RNA (sgRNA), which consists of a scaffold Cas9 binding sequence and ~20 bp nucleotide sequence conferring targeting specificity through complementary base pairing with the DNA target^10^. Cas9 cleaves the target and creates a DNA double-stranded break via activity of its RuvC and HNH endonuclease domains^11^.

CRISPR-Cas9 when applied to a cell population generates a mosaic of cells with varying insertions and deletions^12^ which necessitates clonal expansion of individual cells to obtain a cell line with only the desired gene disruption^13^,^14^. However, the feasibility of using CRISPR-Cas9 for gene deletion in primary ECs, which cannot be clonally expanded^15^,^16^, has not yet been established. A recent method using CRISPR-Cas9 achieved gene deletion following antibiotic selection of clones in cord blood derived endothelial colony forming cells (ECFCs)^17^, which exhibit an endothelial cell-like phenotype but represent a specialized progenitor population of highly proliferative ECs found in umbilical cord blood and which can undergo clonal expansion^18^.

In the present study, we describe a CRISPR-Cas9 approach for gene deletion in primary human ECs without clonal expansion. We used two virus expression systems: EGFP-tagged Cas9 was expressed using an adenoviral vector to avoid genomic integration of the nuclease and sgRNA was expressed using a lentiviral vector. To test the feasibility of the method, we deleted the receptor tyrosine kinase Tie2 gene that regulates vascular barrier integrity at the level of adherens junctions of confluent EC monolayers^19^. We observed that in ECs in which Tie2 was deleted using CRISPR-Cas9 exhibited increased basal endothelial permeability and impaired recovery of permeability following challenge with thrombin, which is known to disrupt endothelial adherens junctions^20^,^21^,^22^. Further we demonstrated by next generation sequencing that the Tie2 mutation was stably retained in primary ECs for up to five passages in the absence of clonal expansion.

## Results

### Co-transduction of lentivirus encoding sgRNAs and adenovirus expressing EGFP-Cas9 in primary endothelial cells

**Figure 1:**
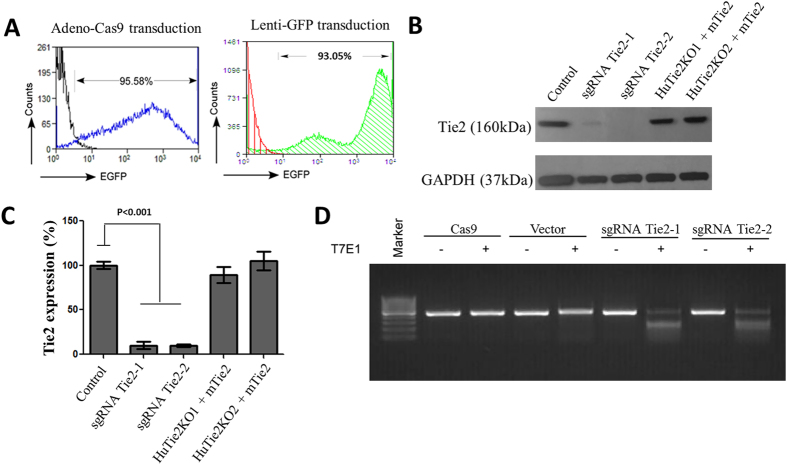
Deletion of Tie2 gene in primary ECs using CRISPR-Cas9. (**A**) Cultured primary HLMVECs were transduced by EGFP-Cas9 adenovirus and sgRNA lentivirus targeting on Tie2 and subjected to flow cytometry analysis of EGFP expression. In a separate study, HLMVECs were transduced by GFP lentivirus and subjected to flow cytometry analysis of EGFP expression as an indicator of lentivirus transduction efficiency. (**B**) Protein expression of Tie2 in vector control and Tie2 knockout HLMVECs induced by CRISPR-Cas9 was determined by immunoblotting. Overexpression of mouse Tie2 in Tie2^−/−^ HLMVECs was determined to assess the ability to rescue Tie2 expression after deletion. The uncropped full-length gels can be found in Supplementary Fig. S1. (**C**) Quantification of Tie2 protein expression from 3 independent experiments. sgRNA Tie2-1 and sgRNA Tie2-2 are CRISPR-Cas9-mediated deletions of Tie-2 at two distinction domains of Tie2 (two different sgRNAs). HuTie2KO1 and HuTie2KO2 + mTie2 represent restoration of Tie-2 expression in cells having undergone Tie-2 deletion. Differences were calculated using one-way ANOVA. P values less than 0.05 are indicated in the graph. (**D**) T7E1 assay detecting mutation on HLMVECs edited by CRISPR-Cas9 with Tie2 sgRNAs as showing a series of bands. Wild-types cells with or without Cas9 or vector only show single bands (negative controls).

Passage 3 HMLVECs were transduced with adenovirus-mediated expression of EGFP-Cas9 at an MOI of 10 as well as a lentivirus expressing a sgRNA targeting the EC receptor kinase Tie2 at an MOI of 10 or a vector control. At 48–72 h post-transduction, confluent EC monolayers were trypsinized and split with a 1:3 ratio, and then co-transduced with a second batch of lentivirus and adenovirus. Transduction efficiency as assessed by flow cytometry was 93% in HLMVECS expressing lenti-GFP and 95% in HLMVECs expressing EGFP-Cas9 (Fig. 1A). We observed Tie2 deletion efficiency of 90% using two distinct sgRNAs (sgRNA Tie2-1 and sgRNA Tie2-2; Fig. 1B,C). Overexpression of mouse Tie2 (mTie2) using adenovirus in *Tie2*^−/−^ HLMVECs restored Tie-2 protein expression (Fig. 1B,C). T7E1 assay was used to confirm the presence of CRISPR-Cas9-induced mutations in HLMVECs following Tie-2 gene deletion (Fig. 1D). ECs transduced with CRISPR-Cas9 and Tie-2 sgRNA displayed a series of bands indicating successful gene editing whereas control cells transduced only with Cas9 or a control vector demonstrated one single band and thus showed no evidence of gene editing (Fig. 1D).

### Next generation sequencing analysis of Tie2 mutation

**Figure 2:**
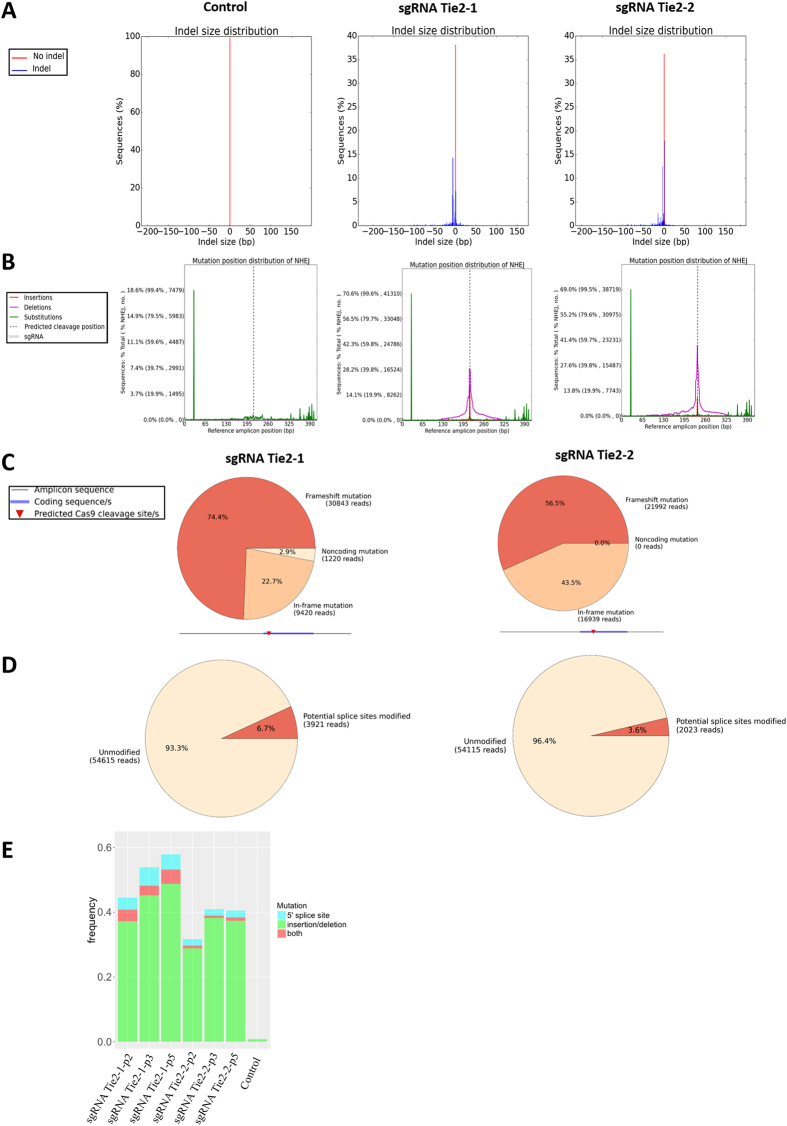
Analysis of CRISPR-Cas9 induced Tie2 gene mutations using next-generation sequencing. (**A**) Indel size distribution of wild-type cells (control) and cells transduced with two different sgRNAs targeting Tie2 (sgRNA Tie2-1, sgRNA Tie2-2). (**B**) NHEJ reads with insertions, deletions, and substitutions were mapped to reference amplicon position. Sequencing/alignment errors (green lines) can be distinguished from indels by their similar positions in all three samples. (**C**) Frameshift mutagenesis profile and predicted Cas9 cleavage site. Unmodified reads are excluded from this analysis. sgRNA Tie2-1 and sgRNA Tie2-2 showed different percentage of reads with mutations from frameshift, in frame, and in noncoding region. (**D**) Predicted impacts on splice sites. Potential splice sites modified refers to the reads in which either of the two introns adjacent to the exon is disrupted. (**E**) The relative contributions of potentially disruptive coding region mutations (indels) and non-coding region mutations (5′ splice site) were quantified across 3 passages. SNPs, in-frame indels, indels less than 5 residues, and 3′ splice site mutations were excluded.

To characterize CRISPR-Cas9 induced mutations in Tie2 locus, we collected DNA-seq data covering the targeted Tie2 exon and flanking introns. The online analysis tool CRISPResso was used to interpret the mutagenesis profiles. We found that the cells transduced with either Tie2 sgRNA (sgRNA Tie2-1, sgRNA Tie2-2) exhibited insertions and deletions (indels) ranging up to 50 bp whereas control cells showed no indels (Fig. 2A). After the reads were mapped to the reference amplicon position, we observed that Tie2-1 sgRNA resulted in indels near the 195 bp site whereas Tie2-2 sgRNA resulted in indels near the 260 bp site (Fig. 2B). The majority of CRISPR-Cas9-induced mutations were located within 10 bp of the target site. As indels comprising a number of nucleotides that is not divisible by three will cause frameshift mutations, we also determined the distribution of frameshift, in-frame, and noncoding mutations (Fig. 2C). The majority of mutations (50–75%) were frameshift mutations whereas in-frame mutations as expected contributed to one-third of the mutations. Non-coding mutations were found in less than 10% of the reads. We also determined the presence of mutations at splice sites since alternate splicing may affect protein levels. Our analysis demonstrated that the cells transduced with either Tie2 sgRNA (sgRNA Tie2-1, sgRNA Tie2-2) had 3–6% splice site mutations (Fig. 2D).

We next evaluated the long-term stability of gene deletion over multiple passages (passages 2, 3, 5). We examined individual high quality (trimmed) reads for potentially disruptive mutations and used cells treated with the vector without a targeting guide sequence as a negative control for any sequence changes due to PCR or sequencing errors. Both Tie-2 sgRNAs induced potentially disruptive mutations in 40–60% of the sequencing reads (Fig. 2E). We classified potentially disruptive mutations as those with 5′ splice site mutations or changes in protein sequence greater than five residues, and found that the proportion of these disruptive mutations was stably maintained up to passage 5 post-transduction. DNA-seq analysis demonstrated that even though CRISPR-Cas9 editing created a mosaic of Tie2 mutations in a population of primary ECs, clonal expansion was not necessary to maintain a stable mutation frequency. This mosaic population of cells was thus used for functional studies even after serial passaging.

### Phenotypic analysis of *Tie2* ^*null*^ ECs

**Figure 3:**
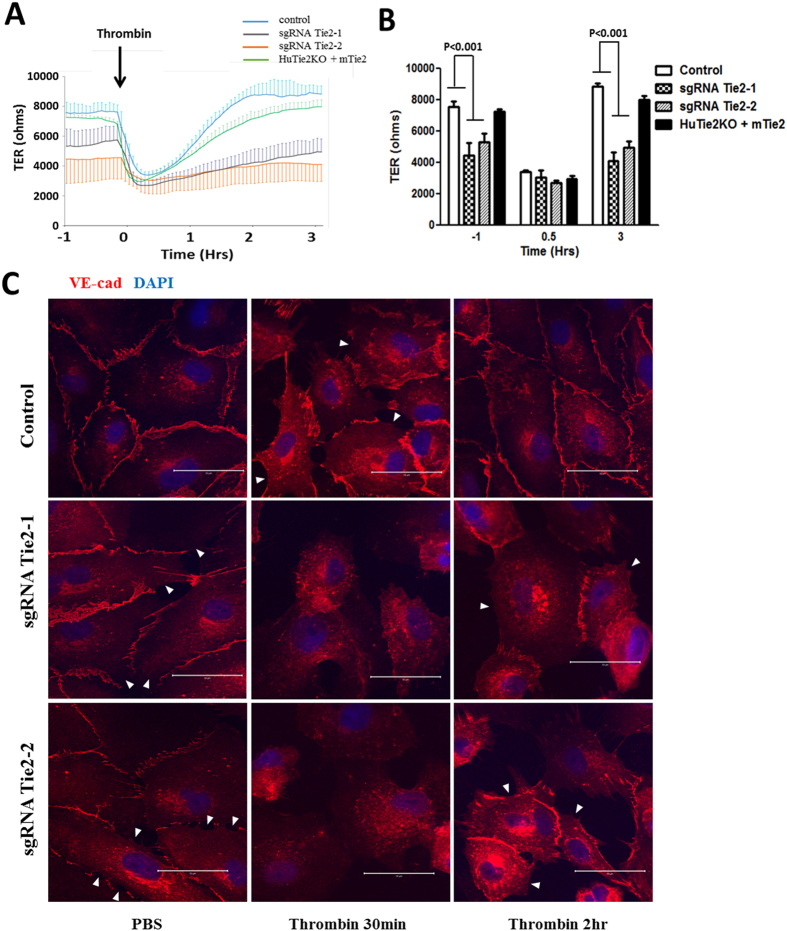
Tie2 deletion by CRISPR-Cas9 in primary ECs increases endothelial permeability and mitigates recovery of permeability in response to thrombin challenge. (**A**) Basal TER and post-thrombin (1 U/ml) TER were studied in confluent control, Tie2-deleted HLMVECs and mTie2 overexpressing cells in which Tie2 had been deleted. Absolute TER values were reduced in both Tie2-deleted groups as compared to control ECs at basal condition. mTie2 overexpression successfully rescued the basal leakiness. (**B**) Quantification of TER values of wild-type (control), transduced cells (sgRNA Tie2-1, sgRNA Tie2-2) and rescued cells (HuTie2KO + mTie2) at basal (−1 h), thrombin-stimulated (0.5 h) and post-recovery (3 h) condition. Differences were calculated using two-way ANOVA. P values less than 0.05 are indicated in the graph. n = 3. (**C**) Serum-starved confluent control or Tie2-deleted HLMVECs were challenged by PBS or 1 U/ml of thrombin, and subjected for VE-cadherin immunostaining at the indicated time-points and analyzed by confocal microscopy. The marked disruption of VE-cadherin junctions seen in wild-type HLMVEC monolayer (control) at the 30 min post thrombin (white arrows) was reversed by 2 h; however, the defective VE-cadherin junctions were present in Tie2-deleted HLMVECs 2 h post-thrombin. White arrows are used to identify areas of adherens junction disruption where neighboring cells lack cell membrane localization of VE-cadherin. Results are representative of 3 independent experiments.

We next examined the function of Tie2 deletion in primary human ECs. We determined whether the Tie2 signaling pathway is indispensable for endothelial barrier integrity at the level of adherens junctions^19^,^23^,^24^. Deletion of Tie2 with either one of the two sgRNAs resulted in persistently increased endothelial permeability when compared to control cells (Fig. 3A,B), demonstrating that deletion of Tie2 signaling in human ECs disrupts barrier function. Importantly, expression of mTie2 in *Tie2*^−/−^ HLMVECs successfully rescued the basal permeability of HLMVECs and restored the endothelial barrier recovery after thrombin challenge (Fig. 3A,B). Immunofluorescence confirmed that Tie2-null ECs exhibited endothelial barrier disruption at adherens junctions whereas control ECs displayed normal cell-cell contact and barrier function (arrow heads) (Fig. 3C). Re-assembly of adherens junctions was completed within 2 h of thrombin stimulation in control ECs whereas junctional barrier restoration was markedly defective in Tie2-null ECs (Fig. 3C).

## Discussion

Although RNA interference (RNAi) is widely used in primary human ECs to study phenotype of vascular and inflammatory signaling pathways, it has important limitations. siRNA-mediated knockdown efficiency is often <70% in ECs and there is a great deal of group to group variability^4^,^25^,^26^. Knockdown also require a host of cumbersome siRNA controls^25^ making it difficult to ascertain specificity of the genes. The transient nature of knockdown and suboptimal transfection efficiencies also pose concerns when using primary ECs. While immortalized EC lines can be used^15^, the transformation of these cells over time compromises their function and does not reflect the physiology of primary ECs.

Here we describe a novel CRISPR-Cas9 method for gene deletion in primary human ECs which overcomes the barrier of clonal expansion for genome editing procedures. Co-transduction of Cas9 and sgRNAs targeting two different sites of the Tie2 gene resulted in a 90% reduction in expression of the Tie2 protein. Sequencing demonstrated that the ECs derived from parent CRISPR-Cas9-edited cells retained gene deletion through 5 passages. Deletion of Tie2 expression resulted in loss of endothelial barrier integrity and defective barrier recovery, consistent with the known Tie2 knockout mouse phenotype^27^,^28^.

The ECs were harvested 48 h after the second batch of viral transduction for protein analysis of Tie2. Since the half-life of Tie2 is around 9 hr in unstimulated ECs and around 3 hr in ECs treated with angiopoeitin-1 (the Tie 2 ligand)^29^, it is likely that most Tie2 proteins would have degraded and the residual Tie-2 protein observed by immunoblotting likely reflected ongoing synthesis of Tie-2 in cells containing the intact Tie2 gene. Therefore, the protein deletion observed in our studies likely resulted from gene disruption rather than dilution of previous existing protein per se.

Our approach employed lentivirus to deliver sgRNA, thus the ECs constitutively expressed sgRNA. We used a lentivirus because it stably transduces both dividing and non-dividing cells^30^,^31^; hence, we were able to achieve high transduction efficiency in primary human ECs. Packaging of only sgRNAs instead of combining both sgRNA and Cas9 into the lentivirus also allowed for smaller construct size and higher viral titer, thereby improving transduction efficiency. Importantly, since adenovirus does not integrate into the host genome^32^ and are thus only transiently expressed for less than 3 weeks^33^,^34^,^35^,^36^, adenoviral delivery of Cas9 reduces the risk of persistent non-specific deletions even if lentivirally delivered sgRNAs continue to be expressed.

A recent study demonstrated gene disruption by CRISPR-Cas9 in highly proliferative EC-like endothelial colony forming cells purified by antibiotic selection, in which the cell number was subsequently expanded^17^. The present study differs significantly in that we used primary ECs, as opposed to stem cell like endothelial colony forming cells, that have limited proliferative capacity in culture and *in situ*^16^,^37^. The present method thus provides a key advantage for gene deleting in untransformed primary human ECs, which may be useful in establishing a pooled stable cell population without antibiotic selection or colony expansion.

Previous studies have demonstrated CRISPR-Cas9 gene editing on primary T cells using either electroporation^38^ or electroporation/adeno-associated virus co-delivery method^39^. However, electroporation itself increases cell membrane permeability^40^, which limits its utility in ECs since a key aspect of EC function is to maintain an intact barrier and electroporation-delivery of CRISPR-Cas9 could compromise physiological studies in adherent ECs. CRISPR-Cas9 has also been delivered to primary airway epithelial cells using a lentivirus^41^; however, this required culture modifications to enhance proliferation of the cells to select cells post-transduction. Such culture modifications could also affect the physiologic state of primary cells. Our novel approach circumvents both clonal expansion and antibiotic selection, thus enabling the study of physiologic functions of the primary ECs following CRISPR-Cas9 gene editing.

Gene editing by CRISPR-Cas9 and other approaches such as Transcription Activator-Like Effector Nucleases (TALEN) create a mosaic of gene edits in a given cell population^12^. T7E1^42^,^43^ and Surveyor Mismatch Cleavage Assays^44^,^45^,^46^ are used to identify CRISPR-induced mutations through recognizing mismatched sites on heteroduplex DNA generated after denaturing and re-annealing of wild-type and mutant strand. Using the T7E1 assay to identify deletion mutations, we found that only cells transduced with Cas9 and sgRNA targeting Tie2 locus demonstrated successful gene editing. However, since the T7EI assay only recognizes indels ≥2 bases which may therefore underestimate the efficacy of CRISPR-Cas9 induced mutagenesis^47^, we performed DNA-Seq to obtain a more sensitive and quantitative assessment of mutations induced by CRISPR-Cas9. We found that the transduced cells have disruptive mutations in 40–60% of the sequencing reads and the mutations were stably maintained up to passage 5 post-transduction. This stable level of mutations over several passages indicates that even though the primary EC population contains a mosaic of cells with varying indels, there does not appear to be any significant change in the ratio of cells that have undergone gene disruption versus those that have not.

We observed a 90% reduction of Tie2 protein expression (Fig. 1B) but next generation sequencing only identified 40–60% potentially disruptive mutations in the sequencing reads (Fig. 2E). One explanation for this discrepancy is that during DNAseq preparation, the amplification and sequencing can miss reads of regions with large deletions. Furthermore, quality control filters in the analysis of next generation sequencing data also discard reads with large deletions, even those which represent true reads with induced mutations rather than mere artifacts. Discarding such reads to improve the quality of sequencing data therefore may underestimate the mutation efficiency. Another possibility is that signaling through the Tie2 receptor may increase expression of the Tie2 protein via a positive feedback loop so that the extent of protein depletion can exceed the predicted loss based on the mutations observed by genome sequencing.

While our approach holds promise for gene editing in fully differentiated human ECs and increases the reliability and safety of the method by using a non-integrating adenoviral vector of Cas9 delivery, there are some limitations. Next generation sequencing showed that CRISPR-Cas9 created a population of mosaic cells. However, our data shows that despite the mosaic nature of the insertions/deletions, the overall cell population exhibited a near-complete disruption of Tie2 protein expression and definitive functional changes, thus confirming that this approach as a valuable tool to address the functional roles of targeted genes with CRISPR-Cas9 mediated gene disruption. Another possible limitation is that our sequence analysis focused on the gene of interest and hence cannot rule out off-target effects in other genes or chromosomes^48^. However, overexpressing mouse Tie2 in Tie2^−/−^ HLMVECs successfully rescued the basal junction leakiness and barrier recovery after thrombin challenge, thus suggesting that the observed functional defects were due to disruption of the solely Tie-2 gene and not due to off-target effects on other EC genes. This potential concern may be further offset by the recent availability of optimized Cas9 variants with enhanced specificity^49^.

In summary, we demonstrate the efficacy of rapid, targeted gene deletion in primary human ECs using CRISPR-Cas9 with a novel dual adenovirus/lentivirus co-delivery system. This method avoided antibiotic selection and clonal expansion and is ideally suited for use in primary cells. Our studies established its utility in ECs but it is likely that this approach can be extended to other primary cells that cannot be clonally expanded. The method constitutes a novel tool for establishing the functional role of genes in primary human cells.

## Methods

### CRISPR-Cas9 system preparation

Guide RNA targeting human Tie2 gene for Cas9-mediated CRISPR disruption were identified using online design software at http://crispr.mit.edu. The two targeting sequences used in this study were: 5′-AGTTAAAGTAGCTGGTAGGA-3′ and 5′-AGCTACTTTAACTATGACTG-3′. DNA oligos were synthesized by IDT and the overlapping PCR products were cloned into pLX-single sgRNA lentiviral vector (a gift from Eric Lander and David Sabatini^14^ and obtained through Addgene as plasmid # 50662). Lentivirus was prepared by co-transfection of lentiviral plasmids with psPAX2 (produced by Didier Trono and obtained through Addgene as plasmid # 12260) and pMD2.G (produced by Didier Trono and obtained through Addgene as plasmid # 12259) packaging plasmids into 90% confluent human 293 T cells (ATCC) cells using Lipofectamine 2000 (Invitrogen) as per manufacturer’s protocol. Lentiviral supernatant was collected at 48 and 72 h post-transfection, concentrated by Lenti-X concentrator (Clontech) as per manufacturer’s protocol, and used to transduce HLMVECs (Lonza CC-2527) at an Multiplicity of infection (MOI) of 10 (measured by Global UltraRapid Lentiviral Titer Kit, System Biosciences) in the presence of 8 μg/ml polybrene (Sigma-Aldrich). At 8 h later, the EGFP-tagged Cas9 adenovirus (Ad-GFP-Cas9, Vector Biolabs # 1901) was also added at an MOI of 10 to transduce HLMVECs. After 2–3 days when HLMVECs reached confluency, the cells were trypsinized and split 1:3 and received a second batch of sgRNA lentivirus and EGFP-Cas9 adenovirus. Cas9 expression was confirmed by flow cytometric analysis of GFP expression in HLMVECs. pWPXL was a gift from Didier Trono (Addgene plasmid # 12257) and is a plasmid with EGFP expression. It was packaged in 293 T cells using the same conditions as the sgRNA plasmid and used in HLMVECs to serve as an indicator of lentiviral transduction efficiency.

### Cell culture

HLMVECs were maintained in culture in a humidified 5% CO_2_ atmosphere at 37 °C in complete Clonetics EGM-2MV BulletKit medium (Lonza, #CC-3202) and 15% fetal bovine serum (Atlanta Biologicals). Cells were thawed at passage 3 and transduced at the same passage.

### Flow cytometry

For transduction efficiency of adenoviral Cas9, this assay was performed on an LSRFortessa (BD Pharmingen) cell analyzer at 48 h post second batch of EGFP-Cas9 adenovirus transduction. For transduction efficiency of lentiviral GFP, this assay was performed on a CyAn ADP (DAKO) cell analyzer at 48 h post second batch of GFP lentivirus transduction. We used 0.05% Trypsin-EDTA to detach HLMVECs, re-suspended them in 0.5 mL PBS/0.2% BSA, and then performed analysis immediately by flow cytometry. Fluorescence compensation was performed with CompBeads (BD Pharmingen).

### Immunofluorescence and confocal microscopy

Control and Tie2^null^ HLMVECs cultured on coverslips were fixed with 4% paraformaldehyde, permeabilized with 0.1% Triton X-100, and probed for VE-cadherin expression to visualize the endothelial barrier (anti-VE-cadherin antibody, Santa Cruz # sc-6458, Donkey anti-Goat IgG (H + L) Secondary Antibody, Alexa Fluor^®^ 594 conjugate, ThermoFisher # A-11058). Images were taken with LSM 880 confocal microscope (Zeiss) and analyzed by LSM510 software.

### Trans-endothelial electrical resistance (TER) junction permeability assay

Endothelial junction integrity was measured by TER assay in the electrical cell-substrate impedance system (ECIS, Applied Biophysics). 50,000 control or Tie2-deleted HLMVECs were seeded in gelatin-coated 8-well array (#8W10E+, Applied BioPhysics) 1 day before the TER measurement. The cells were serum starved and used for TER measurements before and after the expose to 1 U/ml thrombin (GE Healthcare # 27-0846-01). TER changes were monitored using ECIS in real-time at basal level and for 3 h post thrombin as previously described^50^.

### Tie2 mutagenesis analysis

Tie2 knockout was confirmed by immunoblotting for Tie2 (Anti-Tie2/TEK antibody, clone Ab33, Millipore # 05-584; anti-GAPDH antibody, clone 1E6D9, Proteintech # 60004-1-Ig). Western blot pictures were taken using ImageQuant LAS 4000 (GE Healthcare).

### Rescue of Tie2 expression in HLMVECs following Tie-2 deletion

Mouse Tie2 (mTie2) cDNA plasmids was purchased from Sino Biological Inc. (# MG51087-G). To produce the overexpression adenovirus, the mTie2 cDNA was sub-cloned into pShuttle-CMV vector (a gift from Bert Vogelstein^51^ (Addgene plasmid # 16403)) and subsequently recombined into pAdEasy-1 vector (Agilent Technologies) by co-transforming with the BJ5183 cells. After the recombinant Ad plasmid was transformed with XL10-Gold cells, the virus was packaged and amplified in HEK-293AD cells. The harvested mTie2 adenovirus was used to infect Tie2^−/−^ HLMVECs as previously described^52^ and outlined in the AdEasyAdenoviral Vector System User Manual (Agilent Technologies #240009-12).

### T7 Endonuclease I (T7E1) mutation detection assay

Genomic DNA was processed from cultured wild-type cells and CRISPR-edited cells. We used PCR assay to yield cleavage products flanking the Tie2 gene site and verified that only the expected PCR product was synthesized. Heteroduplexes were formed by heating and cooling PCR products in a thermal cycler. Heteroduplexes were incubated with T7E1 (New England Biolabs # M0302S) at 37 °C for 60 min and then the digestion was visualized using agarose gels according to the manufacturer instructions found at www.idtdna.com/.

### Next generation sequencing analysis

DNA extraction on Tie2 gene locus was performed by the Core Genomics Facility (University of Illinois at Chicago). Sequence libraries were generated using a two-step ‘targeted amplicon sequencing (TAS)’ PCR protocol^53^. Amplicons were generated using target-specific primers with 5′-linker sequences (i.e., CS1 and CS2). Subsequently, PCR products were amplified using Fluidigm Access Array primers with sample-specific barcodes. After the second PCR, reactions were purified and normalized using SequalPrep normalization plates (Life Technologies), and sequenced using an Illumina MiSeq instrument, employing V3 chemistry with 2 × 300 base reads. Figure 2A–D: Data was interpreted by CRISPResso (http://crispresso.rocks/) in aspects of aligning to reference amplicon, counting, normalizing, identifying indels, single nucleotide polymorphism (SNP), splice junction and frameshift mutagenesis profiles. Figure 2E: Data was then interpreted independently by adapter trimming and quality filtering using the TrimGalore^54^ wrapper for FastQC and CutAdapt^55^. Overlapping reads were combined with FLASH^56^. Sequence alignment was conducted with the R Bioconductor packages Rsubread^57^ and Rsamtools^58^ against the human Tie2/TEK genomic locus (Ensembl, GRCh38:9:27109141:27230175:1). Only contiguous reads containing the 5′ splice site (TTAAAAGCTTCC, as predicted by SplicePort^59^) and the TEK exon 3 sequence (ENSE00000813529) were considered in evaluating mutation frequencies. Disruptive mutations were considered as those with greater than one nucleotide change in the 5′ splice site, or indels that resulted in a premature stop codon or more than 5 residue changes in the protein sequence. Total reads evaluated for mutations were 36,000 for the vector, and 42,000–52,000 for the TEK sgRNA treatments.

### Statistics

Western blot bands were scanned, and analyzed using Image J software (NIH). All data are expressed as mean ± SD. One-way ANOVA or a t-test were used to determine significance with a p-value threshold set at <0.05 as analyzed by the Prism v5.0 software (GraphPad).

## Additional Information

**How to cite this article**: Gong, H. *et al*. Method for Dual Viral Vector Mediated CRISPR-Cas9 Gene Disruption in Primary Human Endothelial Cells. *Sci. Rep.* **7**, 42127; doi: 10.1038/srep42127 (2017).

**Publisher's note:** Springer Nature remains neutral with regard to jurisdictional claims in published maps and institutional affiliations.

## Supplementary Material

Supplementary Information
