# Supplementary material for: Method for Dual Viral Vector Mediated CRISPR-Cas9 Gene Disruption in Primary Human Endothelial Cells
Source: Sci Rep. 2017 Feb 15;7:42127. doi: 10.1038/srep42127 (PMC5309830; doi:10.1038/srep42127)
Supplement: Supplementary Information [file srep42127-s1.pdf]

Dual Viral Vector Mediated Persistent CRISPR-Cas9 Gene Disruption in Primary Human Endothelial Cells

Supplementary Information

Haixia Gong[1,2†], Menglin Liu[1,2†], Jeff Klomp[1,2], Bradley J Merrill[3, 4],

Jalees Rehman[1,2,5*], and Asrar B. Malik[1,2*]

[1]Department of Pharmacology, [2]The Center for Lung and Vascular Biology, [3]Department of Biochemistry and Molecular Genetics, [4]Genome Editing Core, and [5]Division of Cardiology, Department of Medicine, University of Illinois College of Medicine, Chicago, IL 60612, USA.

[†] contributed equally to the work

For correspondence:

Asrar B. Malik PhD or Jalees Rehman MD,

E-mail: abmalik@uic.edu or jalees@uic.edu

Supplementary Figure Legends

Figure S1. Full length gels for Figure 1B. Immunoblotting of Tie2 protein in vector control, Tie2 knockout HLMVECs induced by CRISPR-Cas9 (sgRNA Tie2-1, sgRNA Tie2-2), and mouse Tie2 overexpression in $Tie2^{-/-}$ HLMVECs (HuTie2KO1 + mTie2, HuTie2KO2 + mTie2,). The pictures of target proteins (chemiluminescence) and protein ladder standards (fluorescence) can only be taken separately by ImageQuant LAS 4000 software. GAPDH was used as the loading control.

Figure S1

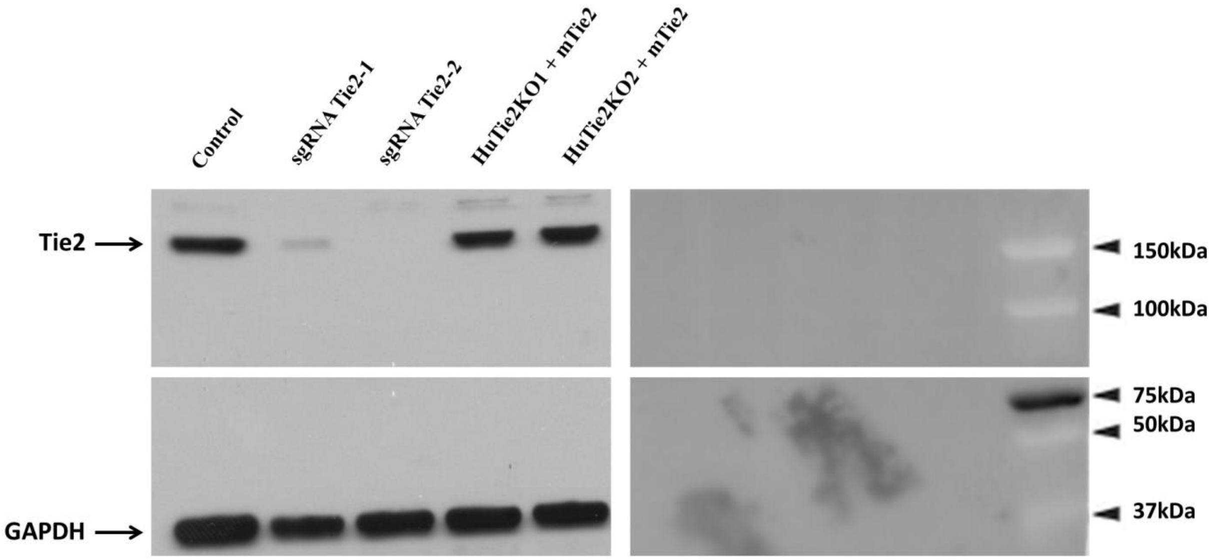
Control
sgRNA Tie2-1
sgRNA Tie2-2
HuTie2KO1 + mTie2
HuTie2KO2 + mTie2
Tie2
GAPDH
150kDa
100kDa
75kDa
50kDa
37kDa
